# Application of RNA interference and protein localization to investigate housekeeping and developmentally regulated genes in the emerging model protozoan *Paramecium caudatum*

**DOI:** 10.1038/s42003-024-05906-2

**Published:** 2024-02-19

**Authors:** Yunyi Gao, Therese Solberg, Rui Wang, Yueer Yu, Khaled A. S. Al-Rasheid, Feng Gao

**Affiliations:** 1 Key Laboratory of Evolution & Marine Biodiversity (Ministry of Education), and Institute of Evolution & Marine Biodiversity, Ocean University of China, Qingdao, 266003 China; 2 Department of Molecular Biology, Keio University School of Medicine, Tokyo, 160-8582 Japan; 3 Human Biology Microbiome Quantum Research Center (WPI-Bio2Q), Keio University, Tokyo, 108-8345 Japan; 4 Zoology Department, College of Science, King Saud University, Riyadh, 11451 Saudi Arabia; 5 Laoshan Laboratory, Qingdao, 266237 China

**Keywords:** RNAi, DNA recombination, Chromosomes, Genetic engineering

## Abstract

Unicellular eukaryotes represent tremendous evolutionary diversity. However, the molecular mechanisms underlying this diversity remain largely unexplored, partly due to a limitation of genetic tools to only a few model species. *Paramecium caudatum* is a well-known unicellular eukaryote with an unexpectedly large germline genome, of which only two percent is retained in the somatic genome following sexual processes, revealing extensive DNA elimination. However, further progress in understanding the molecular mechanisms governing this process is hampered by a lack of suitable genetic tools. Here, we report the successful application of gene knockdown and protein localization methods to interrogate the function of both housekeeping and developmentally regulated genes in *P. caudatum*. Using these methods, we achieved the expected phenotypes upon RNAi by feeding, and determined the localization of these proteins by microinjection of fusion constructs containing fluorescent protein or antibody tags. Lastly, we used these methods to reveal that *P. caudatum PiggyMac*, a domesticated *piggyBac* transposase, is essential for sexual development, and is likely to be an active transposase directly involved in DNA cleavage. The application of these methods lays the groundwork for future studies of gene function in *P. caudatum* and can be used to answer important biological questions in the future.

## Abstract (web summary)

Methods of gene silencing by RNAi and protein localization by microinjection of fusion constructs have been established in the emerging model protozoan *Paramecium caudatum*. These methods lay the groundwork for future studies of gene function.

## Introduction

Over the course of evolution, unicellular eukaryotes have not only become an important part of the bulk biodiversity on Earth, but have also evolved numerous unique molecular mechanisms, making them attractive as model systems^1–5^. Ciliates are a morphologically diverse and highly differentiated group of unicellular eukaryotes whose studies have led to numerous remarkable discoveries throughout the years^6–11^, perhaps the most famous of which is the discovery of telomeres and telomerase in *Tetrahymena thermophila*^12–14^. Despite extensive research, genetic engineering techniques are still only available in a few model species and thus cannot represent the breadth of ciliate diversity. This remains a major obstacle in our progress toward understanding the molecular mechanisms underlying this diversity.

The ciliate *Paramecium* has been used as a model to investigate many diverse scientific questions over the years, greatly facilitated by the availability of molecular methods in the model organism *Paramecium tetraurelia*^15–18^*. Paramecium*, like all ciliates, has two functionally distinct nuclei: the diploid germline nucleus (micronucleus, MIC) and the polyploid somatic nucleus (macronucleus, MAC)^19,20^. During sexual reproduction, the germline genome undergoes large-scale DNA elimination and reorganization to form a new somatic genome through a series of genome rearrangement processes^21–23^. Studies into these processes have provided insights into transposon silencing, epigenetics and various chromatin-related processes and have made many contributions to cell and molecular biology^16,24,25^.

**Fig. 1:**
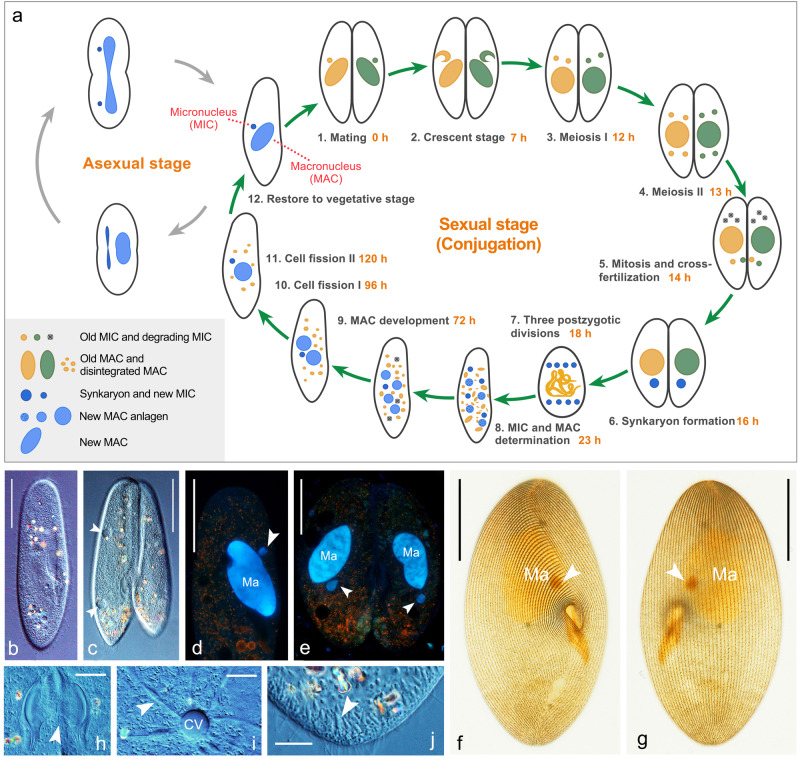
Life cycle and photomicrographs of *Paramecium caudatum*. **a** Life cycle of *Paramecium caudatum*. Gray arrow circle: vegetative (asexual) stage. Green arrow circle: conjugation, the sexual stage of the life cycle. **b**, **c** Ventral views of a vegetative cell and a conjugated pair in vivo. Arrowheads mark two contractile vacuoles. **d**, **e** A vegetative cell and a conjugating pair showing the macronuclei (Ma) and micronuclei (arrowheads) after staining with Hoechst 33342 and acridine orange. **f**, **g** Ventral and dorsal views of a cell to show the ciliature after silver carbonate impregnation. **h** Detailed images of the oral membranes in a conjugated pair. The arrowhead points to the fusion of plasma membranes. **i** Detailed images of contractile vacuoles (CV) and collecting canals (arrowhead) visualized by DIC. **j** Spindle-shaped trichocysts (arrowhead) visible beneath the plasma membrane. Scale bars: 50 μm in **d**–**g**; 20 μm in **h**–**j**.

In addition to *P. tetraurelia*, another well-known representative of the genus *Paramecium*, *P. caudatum*, also has protocols for cultivating strains and inducing conjugation^26,27^ (Fig. 1). *P. caudatum* diverged from the *P. aurelia* complex before the two most recent whole-genome duplications (WGDs)^28,29^, which allowed it to have fewer homologous genes and a smaller MAC genome size (~30 Mb in *P. caudatum* vs. ~70 Mb in *P. tetraurelia*). Despite the smaller MAC genome, *P. caudatum* has the largest MIC genome size reported in ciliates (approximately 1,600 to 5,500 Mb in *P. caudatum* vs. 108 Mb in *P. tetraurelia*, 157 Mb in *T. thermophila* and ~500 Mb in *Oxytricha trifallax*)^30–32^. With such enormous differences in the size of the two genomes, studies in *P. caudatum* are likely to provide insight into the process of genome rearrangements. However, genetic engineering techniques have not yet been reported in *P. caudatum*, greatly hampering such investigations.

In the present study, we sought to develop important tools to facilitate research on *P. caudatum* by adapting gene manipulation methods widely used in *P. tetraurelia*, including silencing by RNA interference (RNAi) and protein localization by microinjection of transgenes encoding fusion proteins with fluorescent tags^24,33–35^. As a proof-of-concept, we first chose two house-keeping genes to test the applicability of the methods: the non-discharge gene *ND7* and the largest subunit of DNA-directed RNA polymerase II (*RPB1*), which have both been characterized in *P. tetraurelia*. Once the methods had been established, we further adapted them for developmentally regulated genes by silencing the domesticated *piggyBac* transposase *PiggyMac* (*PGM*), a key player in the DNA elimination process in *P. tetraurelia*^36^. These results demonstrate that knock-down of both vegetatively expressed genes (*ND7* and *RPB1*) and developmentally expressed genes (*PGM*) can be successfully achieved through RNAi by feeding in *P. caudatum*. Moreover, microinjection of GFP and FlagHA-tagged fusion constructs yielded fluorescence signals consistent with the expected protein localizations of the genes of interest. Taken together, our study successfully established essential genetic manipulation methods in *P. caudatum*, which lays the foundation for future studies of gene function in this model protozoan.

## Results

### Knockdown of *ND7* by RNAi generates exocytosis-deficient cells

The non-discharge (ND) gene *ND7* is involved in a specialized pathway for regulated secretion in *Paramecium*, required for exocytotic membrane fusion and trichocyst release^37^. In response to various stimuli such as treatment with picric acid or dilute acetic acid, trichocysts can be excreted and their length eightfold expanded, allowing simple and rapid assessment of phenotypic defects for quantitative evaluation^38–40^. The *ND7* gene has been studied in *P. tetraurelia* for many years^37,41^, and is frequently used as a positive control to test the efficiency of gene silencing^36,38^. Therefore, we decided to first test the applicability of RNAi in *P. caudatum* using this gene.

**Fig. 2:**
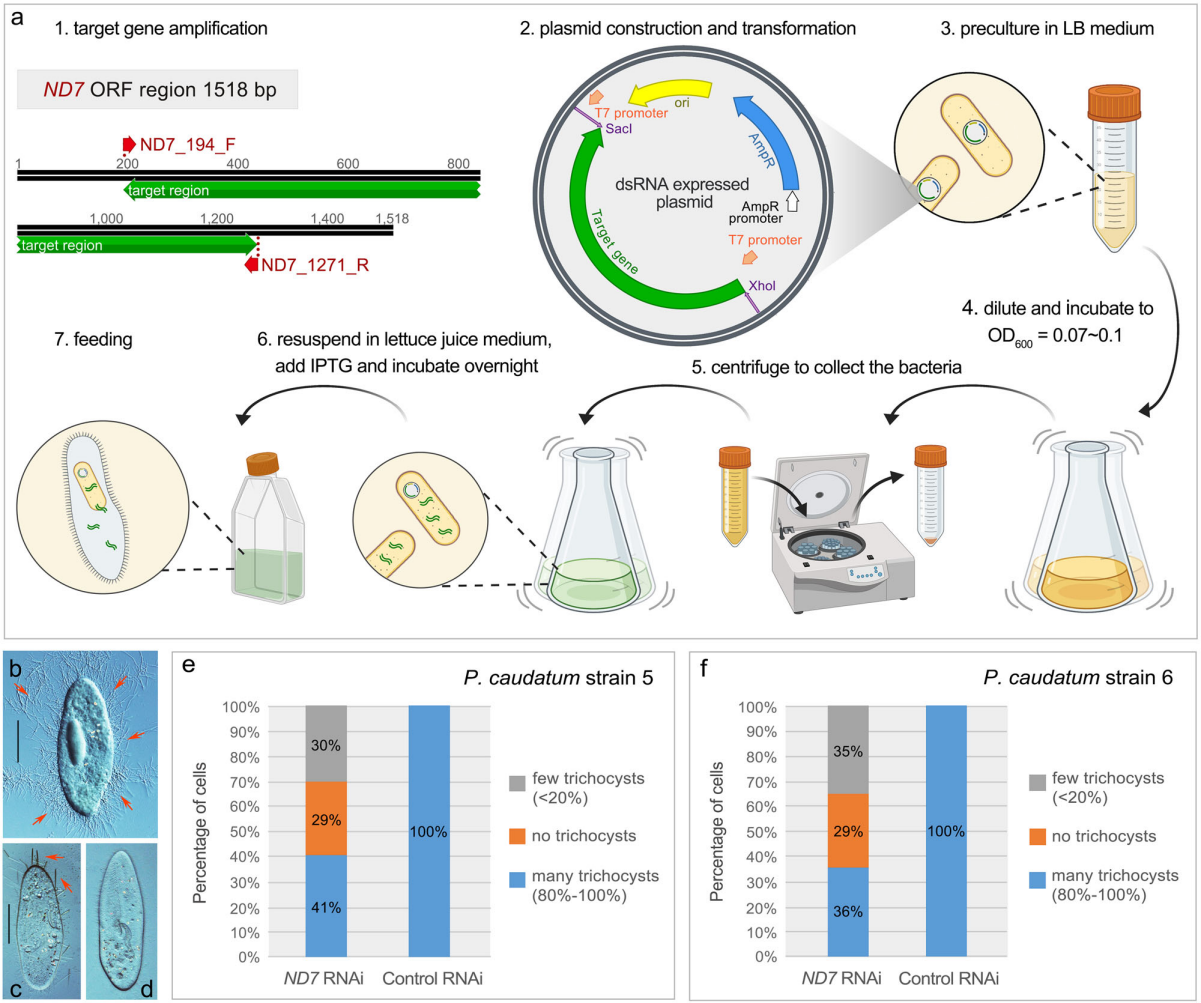
RNAi protocol and *ND7* silencing in *Paramecium caudatum*. **a** 1. The *ND7* open reading frame and the region used for RNAi. 2–7. Schematic diagram of the RNAi method in *P. caudatum*. **b**–**d** Representative images of control and *ND7* RNAi cells upon treatment with 20% acetic acid. **b**, **c** Arrows point to secreted trichocysts in control RNAi (**b**, many trichocysts) or *ND7* RNAi (**c**, few trichocysts). **d** A representative image of a cell with no trichocyst discharge after *ND7* RNAi. **e**, **f** Stacked bar charts of the proportion of cells with trichocyst discharge in *P. caudatum* strains 5 and 6, *n* = 80 cells. The bars are divided into three categories based on the amount of trichocyst discharge, shown in different colors. Representative images of each category are shown in **b**–**d**. Scale bars: 50 μm.

Only one *ND7* gene sequence was identified in the MAC genome of *P. caudatum* using the publicly available Paramecium database^42^. However, PCR amplification yielded two distinct *ND7* gene sequences (92.8% nucleotide identity) in both strains tested, with the majority of the differences between them being single base substitutions (Supplementary Fig. 1). It is possible that the ‘duplicate’ *ND7* genes in our *P. caudatum* strains are alleles. Because *P. caudatum* cannot undergo autogamy, if the zygote that developed into MACs are heterozygotes, it will result in a heterozygous MAC genome. The discrepancy between the MAC genome assembly (a single *ND7* sequence) and the recovery of two *ND7* sequences following PCR amplification may be that the second *ND7* gene was deleted as a redundancy during MAC genome assembly. The other explanation is that there is a high gene copy number of the MAC genome in *P. caudatum* (~3400n), the low copy number of the second *ND7* gene may not be well sequenced in the MAC genome. We therefore constructed RNAi plasmids containing targets for each gene to efficiently silence the expression of *ND7*. The targets for each gene were cloned between two inverted T7 promoters of the L4440 vector and transformed into a RNaseIII-deficient *E. coli* strain to allow the production of dsRNAs upon IPTG induction (Fig. 2a). *P. caudatum* cells were then fed with dsRNA-expressing *E. coli* containing a mixture of both constructs (1:1). Control cells were fed HT115 bacteria with empty L4440 plasmids. To evaluate the efficiency of *ND7* RNAi, we treated both *ND7* silenced and control cells with 20% acetic acid after continuous feeding for 5 days to assess their ability to discharge trichocysts. All control cells secreted trichocysts, which appeared as a dense halo surrounding the cells (Fig. 2b). In the *ND7* RNAi culture, only around 40% of the cells were able to secrete trichocysts (Fig. 2c, d), whereas the remaining 60% of cells had no or very few trichocysts discharged (Fig. 2e, f). The *ND7* silencing was performed three times, with similar results obtained each time, demonstrating a high level of reproducibility. Hence, RNAi by feeding can be applied in *P. caudatum* to generate exocytosis-deficient cells.

### Knockdown of *RPB1* by RNAi is fatal to the cells

**Fig. 3:**
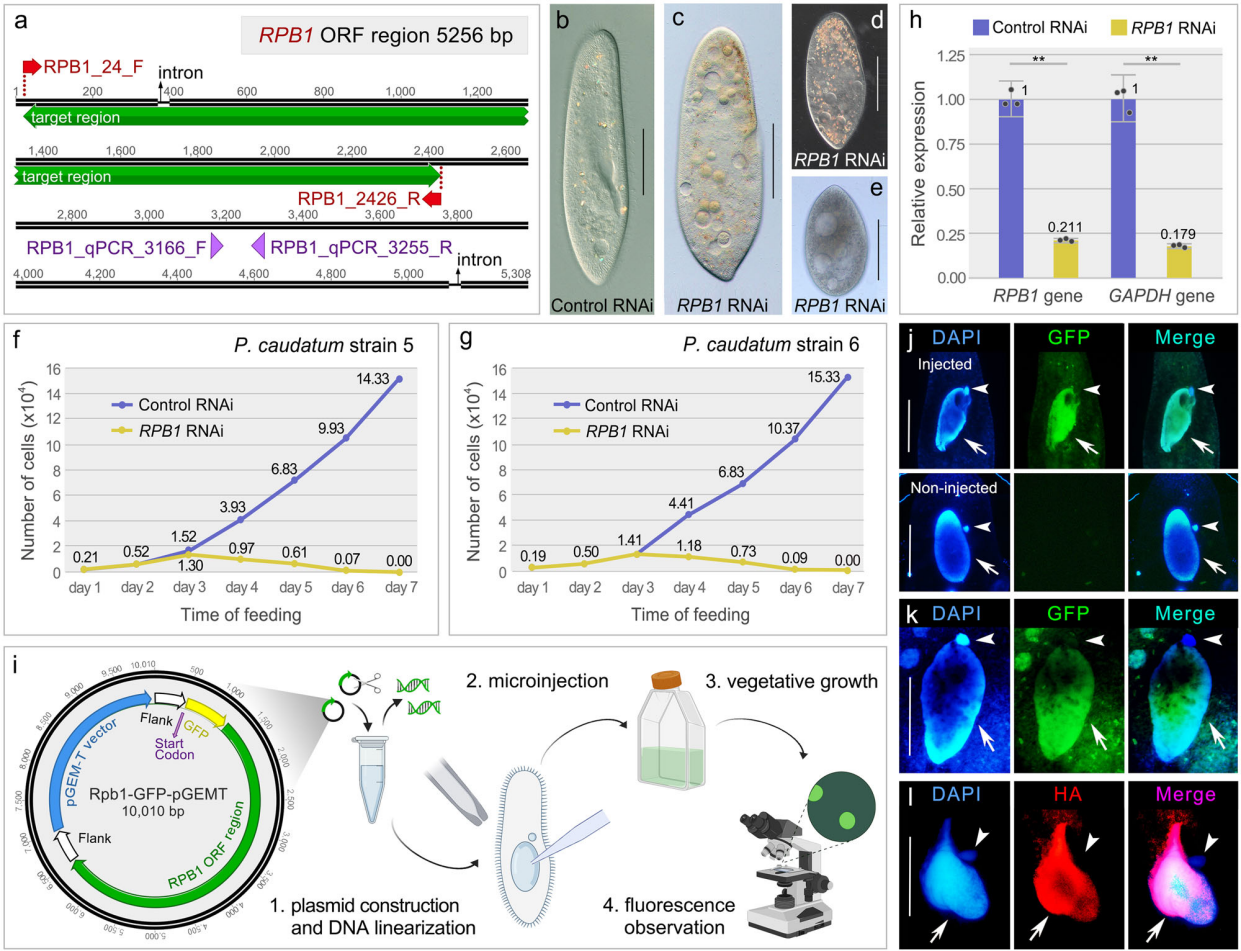
*RPB1* silencing and protein localization in vegetative cells. **a** The *RPB1* open reading frame and the region used for RNAi (green). Double lines represent exons and single lines introns. The primers used for qPCR analysis are marked with purple arrowheads. **b** Photomicrograph after 7 days of control RNAi. **c–e** Photomicrographs after 4–7 days of *RPB1* RNAi. **f**, **g** Growth rates of *P. caudatum* strain 5 and 6 after control RNAi (blue) or *RPB1* RNAi (yellow). The x-axis shows the time points, and the y-axis indicates the number of cells. Numbers on the lines show the cell number on each day. **h** *RPB1* and *GAPDH* mRNA expression levels measured by RT-qPCR and normalized to small subunit rRNA expression in control (blue) and *RPB1* RNAi cells (yellow). The x-axis represents *GAPDH* and *RPB1* mRNAs, respectively; and the y-axis shows the relative mRNA expression levels. The dots represent the relative quantification of three replicates. Numbers show the mean relative quantification. The error bars show the relative quantification minimum and maximum. The LSD test in one-way analysis of variance was used to analyze the significance. Two asterisks indicate *p* < 0.01. **i** Schematic diagram of protein localization method in *P. caudatum*. **j–l** Localization of Rpb1 by fluorescence microscopy. **j** Localization of Rpb1-GFP 2 days after microinjection. **k** Localization of Rpb1-GFP one month after microinjection. **l** Localization of Rpb1-HA after immunofluorescence staining. Arrows indicate the MACs and arrowheads indicate the MICs. Scale bars: 50 μm.

Since we were able to induce an exocytosis-deficient phenotype upon *ND7* RNAi, we decided to further test the applicability of this method by knocking down an essential housekeeping gene. We chose the DNA-directed RNA polymerase II subunit Rpb1, the largest subunit of RNA polymerase II that plays important roles in transcribing messenger RNAs (mRNAs), most small nuclear RNAs (snRNAs) and microRNAs^43^. Only one *RPB1* gene sequence was identified in the macronuclear genome of *P. caudatum*, which was further confirmed by PCR amplification in both strains. To generate the RNAi plasmid, a fragment of the *RPB1* gene was selected as the RNAi target and plasmid construction and silencing were carried out as described (Figs. 2a and 3a). Cells fed with HT115 bacteria containing empty L4440 plasmids were used as controls. After three days of silencing, while the control cells which were fed with *E. coli* containing an empty L4440 plasmid were phenotypically normal (Fig. 3b), the shape of the *RPB1* RNAi cells became aberrant under the same growth conditions (Fig. 3c–e). On the fourth day, many vacuoles appeared in the cells (Fig. 3c). The vacuoles grew larger over time, accompanied by longitudinal contraction of the cells (∼50% shorter in length, Fig. 3d, e), followed by death within seven days of feeding.

At the same time, the division rates of the cells were plotted. There was no obvious difference between *RPB1* RNAi and control cells in the first three days of feeding (Fig. 3f, g). With continuous silencing, the number of *RPB1* RNAi cells began to decline from the fourth day and the cells eventually died within seven days. The division rates of strains 5 and 6 had a similar trend, indicating that *RPB1* RNAi had similar effects on both strains (Fig. 3f, g). We repeated the experiment three times under the same conditions and observed the same phenotypes, demonstrating that the results are highly reproducible.

After five days of silencing, we also quantified the silencing effect by measuring the expression level of *RPB1* using reverse transcription and quantitative PCR (RT-qPCR). We used the small subunit rRNA (transcribed by RNA polymerase I and not affected by *RPB1* silencing) as an internal control to normalize the samples, and each reaction was performed three times in parallel. Compared to the control, the expression level of *RPB1* was significantly lower in the *RPB1* RNAi culture (*p* = 0.005, Fig. 3h). Moreover, we examined the expression level of the *GAPDH* gene, which is transcribed by RNA polymerase II, to determine whether *RPB1* RNAi affected the expression of other genes. Similarly, the expression level of *GAPDH* was significantly lower in the *RPB1* RNAi culture than in the control (*p* = 0.008, Fig. 3h). These results suggest that *RPB1* was successfully knocked down, and the loss of this protein affected the expression of other genes.

### Rpb1 localizes in the transcriptionally active MAC

Since the goal of this study was to establish important methods for the study of protein function in *P. caudatum*, we next sought to express tagged fusion proteins that would allow further investigation into protein function. The localization of proteins provides key insight into their function, and we therefore first designed GFP-tagged constructs for localization studies. To do so, the Rpb1 protein was tagged with a codon-optimized GFP^44^, inserted after the start codon ATG of the *RPB1* ORF (Fig. 3i). The construct, containing GFP, the *RPB1* ORF and putative regulatory regions of the *RPB1* gene, was linearized and microinjected into the MAC of vegetative cells. Two days after transformation, a fluorescent signal was detected in the MACs of injected cells, but not in non-injected control cells, confirming the expression of the injected construct and subsequent import of the protein product into the nucleus (Fig. 3j). Of note, we detected no GFP signal in the transcriptionally inactive MIC, consistent with selective import of the protein into the transcriptionally active nucleus only, as expected for an RNA Polymerase II subunit. Moreover, the injected cells displayed no phenotypic aberrations nor impaired growth, suggesting that the protein products do not cause dominant-negative effects to the cells. To assess the expression and maintenance of the injected construct after many cell fissions, we observed the GFP signal after prolonged culturing. GFP fluorescence could still be observed in almost all descendants after 40 cell fissions, confirming stable expression of the injected construct (Fig. 3k).

Although GFP-tagging has become the standard for protein localization studies in ciliates, GFP is a rather large tag and is not as commonly used for protein purification or other assays into protein function. Several studies have successfully utilized the smaller Flag-HA tag (33 amino acids containing both Flag and HA tags) for a variety of such assays^25,45,46^. We therefore also generated Flag-HA fusion constructs by inserting Flag-HA after the start codon ATG of the *RPB1* gene. Immunofluorescence after Rpb1-Flag-HA microinjection showed that the fusion protein could be detected in the MAC, consistent with the localization of the GFP-fusion (Fig. 3l). Similarly to the Rpb1-GFP injectants, we observed no phenotypic aberrations nor impaired growth. Taken together, these experiments demonstrate that transformation by microinjection can be a powerful tool for the in vivo study of protein function in *P. caudatum*.

### Knockdown of *PGM* by RNAi inhibits IES excision and is lethal to sexual progeny

**Fig. 4:**
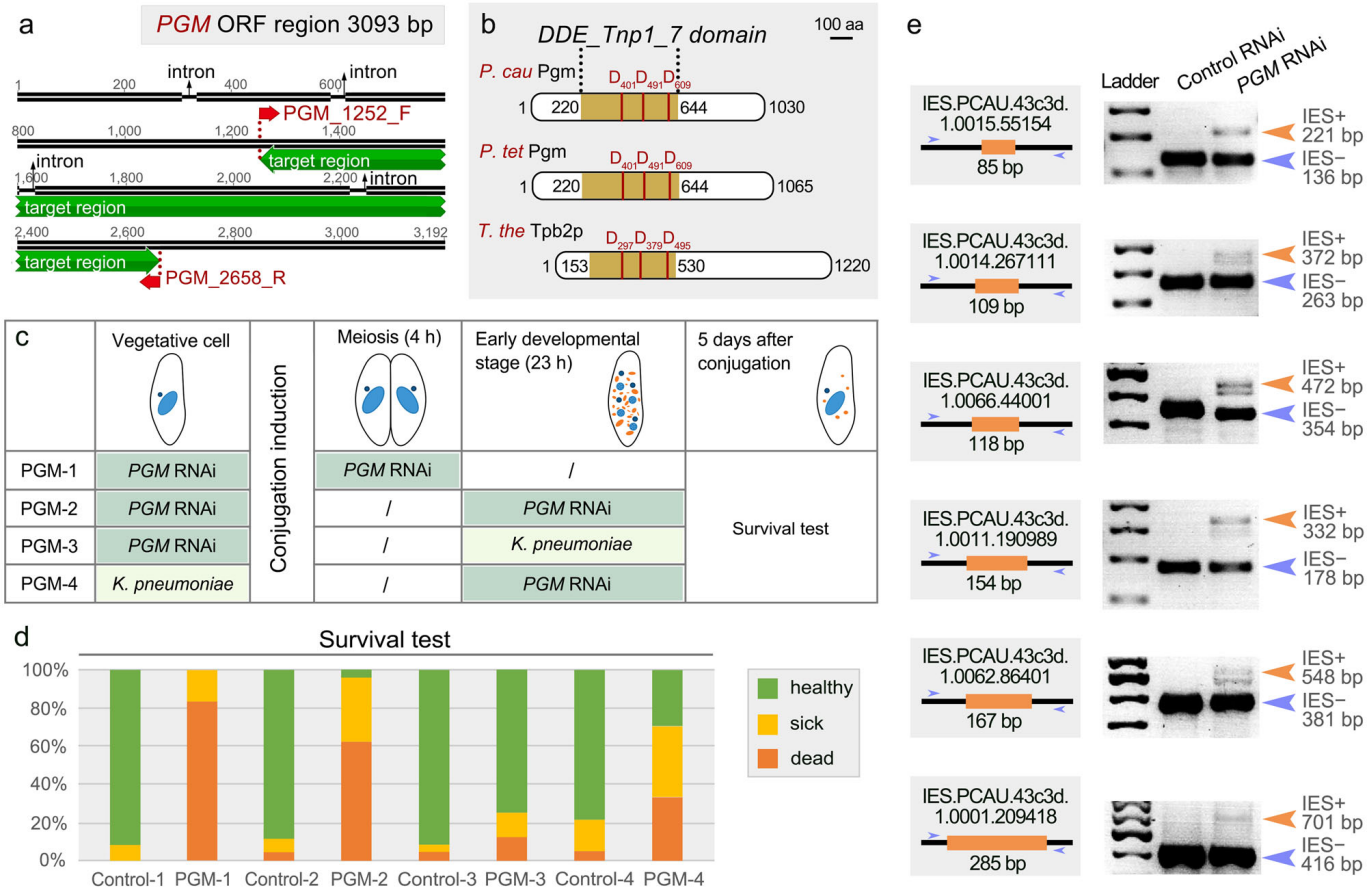
Structure of the Pgm protein and phenotype after *PGM* depletion. **a** The *PGM* open reading frame and the region used for RNAi (green) **b** Domain organization of domesticated *piggyBac* transposases in *Paramecium caudatum* (Pgm, first) *Paramecium tetraurelia* (Pgm, second) and *Tetrahymena thermophila* (Tpb2p, third). The DDE_Tnp_1_7 domain was predicted by InterPro (https://www.ebi.ac.uk/interpro/) and is shown as yellow boxes. Conserved catalytic residues (D residues) are indicated by red vertical bars. **c** Table describing the four *PGM* RNAi experiments. Each row in the table represents a different experimental group and each column represents a different silencing time. Green table cells represent feeding with *PGM* silencing medium, and yellow represents feeding with *K. pneumoniae* (control) medium. **d** Survival tests after silencing, assessed by growth rate compared to wild type. The x-axis denotes the silencing condition as described in c. The y-axis denotes the percentage of cells examined, *n* = 24 cells. Green (Healthy), normal growth as wild type; yellow (sick), slow-growing; orange (dead), death after less than three cell divisions. **e** IES retention assessed by PCR on DNA extracted after completion of sexual development in control and *PGM* silenced cells. Orange arrows show the size of bands with IES retention (IES+), and blue arrows show the size of bands without IES retention (IES−).

The work described so far established methods for the study of protein function during vegetative growth in *P. caudatum*. However, ciliates also undergo complex genome rearrangement processes to remove transposable elements and ancient transposon remnants known as Internally Eliminated Sequences (IESs) during sexual development^23^. Due to a lack of suitable methods, little is known about this process in *P. caudatum*. Therefore, we further tested the applicability of RNAi to silence the expression of developmentally regulated genes during sexual processes in *P. caudatum* and chose the *PGM* gene for this purpose. PiggyMac (Pgm) is a domesticated *piggyBac* transposase which eliminates germline-specific DNA (including transposons, repetitive sequences and IESs) during sexual processes in *P. tetraurelia*^36^. One *PGM* gene (Fig. 4a) was identified in the macronuclear genome of *P. caudatum*^42^. This gene harbors an intact DDD catalytic triad, a characteristic of active *piggyBac* transposases, similarly to the domesticated transposases in *P. tetraurelia* and *T. thermophila* (Fig. 4b and Supplementary Fig. 2). Therefore, we hypothesized that *PGM* may perform a similar function in DNA elimination in *P. caudatum*. In *P. tetraurelia*, *PGM* is exclusively expressed during sexual development^36^. Therefore, we first designed multiple experiments to determine the best way of silencing developmentally regulated genes (Fig. 4c). Vegetative cells of both mating types grown in silencing medium for 4 days were mixed, and stable mating pairs (PGM-1) or exconjugants (PGM-2) were individually transferred to new wells containing the same silencing medium to monitor the survival of exconjugants. On the basis of the conserved role of domesticated transposases in other ciliates, we used survival rate as a readout of silencing, as the depletion of this protein is likely to cause retention of germline-specific sequences followed by death of sexual progeny. Indeed, continuous silencing of *PGM* throughout vegetative growth and subsequent conjugation stages led to strong lethality in sexual progeny (Fig. 4d). In parallel, we mixed both mating types grown in silencing medium and transferred exconjugants to *Klebsiella pneumoniae* (*K. pneumoniae*) medium (PGM-3). However, this silencing had little to no effect on the survival of sexual progeny (Fig. 4d). We also performed the opposite experiment, by mixing both mating types grown in *K. pneumoniae* medium and transferring exconjugants to silencing medium (PGM-4). In this condition, 30% of the exconjugants survived (Fig. 4d). For each experimental condition, we also fed cells with HT115 bacteria containing empty L4440 plasmids instead of the silencing medium as controls, while maintaining the rest of the culture conditions the same as the *PGM* silencing. All experiments were repeated three times and the same phenotypes were observed each time, demonstrating that the results are highly reproducible. Taken together, these experiments demonstrate that the strongest phenotype is achieved when cells are fed with silencing medium during both vegetative growth and subsequent conjugation stages (Fig. 4d). Additionally, we extracted genomic DNA from cells following one of the strongest silencing conditions (PGM-2) and performed IES retention PCRs using primers flanking a random set of IESs^30^ (Supplementary Table 1). As expected, depletion of *PGM* by RNAi resulted in IESs retention, suggesting that Pgm in *P. caudatum* is involved in DNA elimination (Fig. 4e).

### Pgm localizes in the developing new MACs

**Fig. 5:**
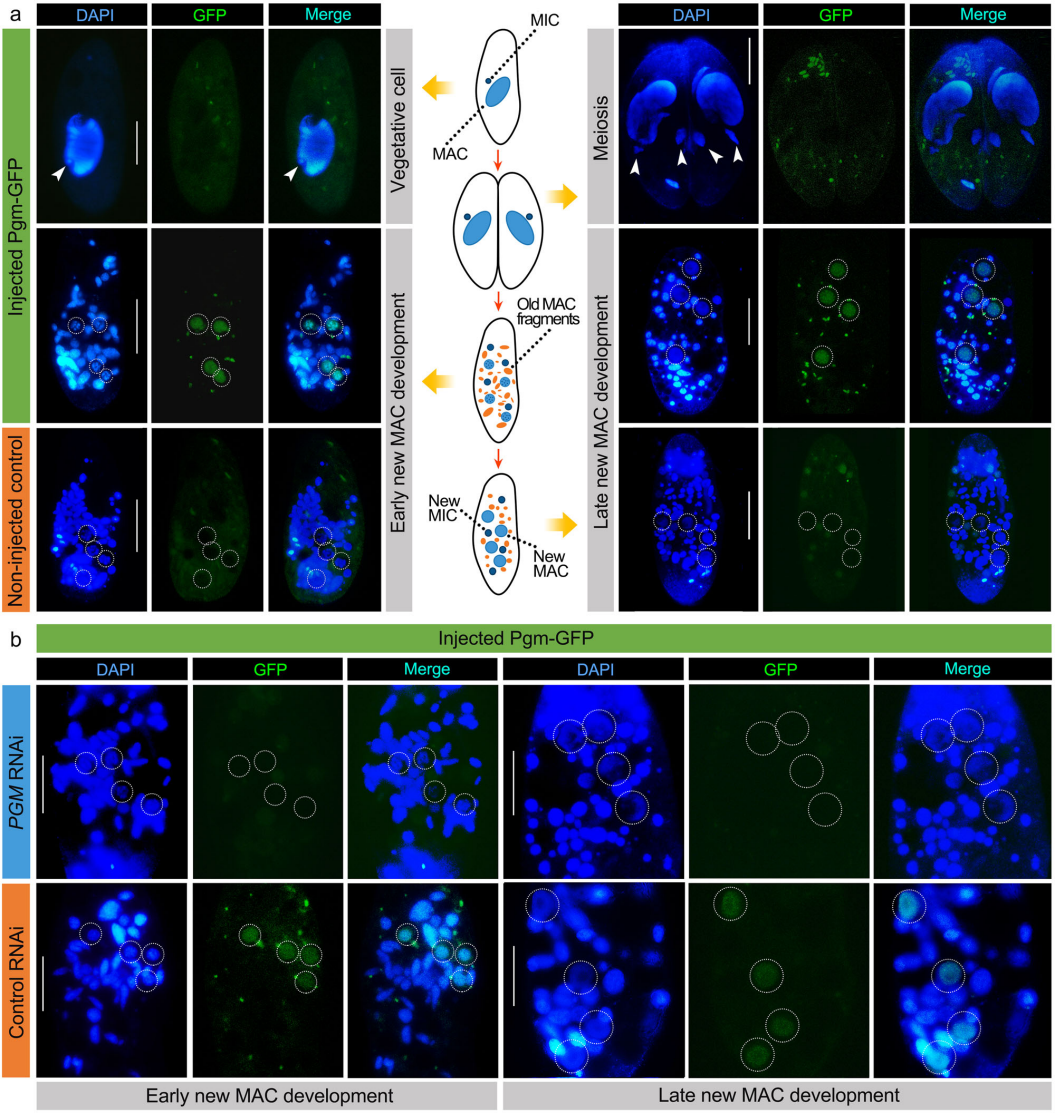
Localization of Pgm-GFP. **a** Localization of Pgm-GFP in vegetative cells and during sexual development (conjugation). DAPI is used as a DNA stain. Arrowheads point to MICs and dashed white circles indicate developing new MACs during early to late new MAC development. **b** Localization of Pgm-GFP in *PGM* or control RNAi cells during new MAC development. Dashed white circles indicate developing new MACs. Scale bars: 50 μm.

In *P. tetraurelia, PGM* is exclusively expressed during sexual development and is required for IES elimination and survival of sexual progeny^36^. Although no expression profiles are available for *P. caudatum*, the IES retention phenotype and close relationship to *P. tetraurelia PGM* suggests a development-specific expression. To confirm this hypothesis and further test the applicability of transformation as a means to generate fusion proteins of development-specific targets, we generated and injected a GFP-tagged Pgm construct into the MAC of vegetative cells. The injected cells displayed no impaired growth and conjugation could be initiated normally, producing viable progeny. We detected no GFP fluorescence in vegetatively growing injectants, nor in non-injected control cells in any stage (Fig. 5a). During new MAC development, the fusion protein localized in the developing new MACs specifically (Fig. 5a). Hence, transformation by microinjection of development-specific constructs allows development-specific expression of fusion proteins. Importantly, this localization is consistent with an involvement in DNA elimination and the observed IES retention phenotype upon *PGM* depletion.

Furthermore, we performed *PGM* RNAi on both strains of *P. caudatum* using the PGM-2 method after transforming cells with the Pgm-GFP fusion construct. As expected, the GFP signal disappeared from the new MACs upon *PGM* RNAi, further demonstrating the feasibility of the RNAi method (Fig. 5b).

## Discussion

Despite the vast diversity of ciliates, genetic engineering techniques are still only available in a few model species^33–35,47–52^. Here, we aimed to expand this list by adapting common gene manipulation techniques for use in *Paramecium caudatum*, to provide researchers with the tools required for further study of gene function in this organism. We adapted the method of gene knockdown by RNA interference first, since this method has become one of the most widely used tools for loss-of-function studies in ciliates and has been well-established in several species including *Paramecium tetraurelia* and *Paramecium bursaria*^33,53,54^, *Euplotes*^55^, *Oxytricha*^50^, *Stylonychia*^56^, *Stentor*^57,58^ and *Spirostomum*^59^. We also adapted the method of microinjection as a means to transform cells with fusion constructs for protein localization studies. Taken together, we successfully implemented key genetic engineering techniques for use in *P. caudatum*, including silencing by RNAi and transformation by microinjection, to study the role of both housekeeping and developmentally regulated genes.

The silencing phenotypes we observed in this study were all consistent with the previously reported functions of Rpb1, ND7 and Pgm in *P. tetraurelia*^36,37,43^. Nevertheless, we found that the efficiency of silencing by RNAi seems to be variable between genes and species. Silencing of essential genes, such as *RPB1*, resulted in almost 100% abnormal or dead cells, while the silencing of *ND7* resulted in a trichocyst discharge defect in only ~60% of cells. Hence, the silencing effect of *ND7* RNAi appears to be less than *RPB1* RNAi. One explanation may be that even very low expression of *ND7* is enough to discharge trichocysts. Alternatively, this may be due to redundancy since two distinct *ND7* gene sequences (92.8% nucleotide identity) are present in the MAC genome. Interestingly, we found that silencing of developmentally regulated genes cannot be achieved by RNAi during vegetative growth alone, which may suggest that the dsRNAs are depleted before conjugation. To overcome this limitation, we designed multiple experiments to increase the amount of dsRNA in exconjugants and improve the silencing efficiency of developmentally regulated genes. Although RNAi could be readily applied to a variety of different genes using our approach, these experiments suggested that the efficiency of RNAi may vary depending on the target gene in *P. caudatum*.

We also noted that the efficiency of RNAi in *P. tetraurelia* appears to be higher than that in *P. caudatum*. In *P. tetraurelia*, a single day of RNAi by feeding can successfully silence the expression of developmentally regulated genes, without the need of additional feeding following sexual processes^33,36^. This is in stark contrast to what we found for *P. caudatum*. As mentioned above, efficient silencing of developmentally regulated genes (e.g., *PGM* in the present study) in *P. caudatum* requires feeding prior to as well as following sexual processes. This suggests that knockdown efficiency is variable between species. One reason for the lower RNAi efficiency may be the higher copy number of the MAC genome in *P. caudatum* than *P. tetraurelia* (~3400n vs ~800n)^60^. A similar situation has also been found in insects, where some species are highly susceptible to RNAi while others need large amounts of dsRNA to elicit even a minor knockdown response^61–63^. The variability between and within species may be influenced by various factors such as different efficiencies or activities in response to RNAi mechanisms^64,65^, limited absorption of dsRNA, or dsRNA degradation in vivo^66,67^. Regardless of the underlying reason, it is clear that it is necessary to increase the concentration of dsRNA and providing fresh RNAi bacterial solution in a timely manner to achieve an efficient RNAi silencing in *P. caudatum*.

In terms of gain-of-function study, previous studies in *P. tetraurelia* have shown that plasmid DNA microinjected into the MAC can be de novo telomerized and replicated independently, resulting in persistent high copy numbers of microinjected DNA during vegetative growth^34,35,68^. Similarly, DNA containing GFP microinjected into the MAC of *P. caudatum* resulted in fluorescence that could be maintained for up to 20 fissions post-injection^44,69^. However, Takenaka et al. (2002) reported that only 10–20% of descendants have the fluorescent signal after about 30 to 40 fissions^44^. Here, we demonstrated that the Rpb1-GFP fusion was still detectable in nearly all descendants after 40 fissions post-injection (Fig. 3k). These results provide further evidence that microinjected plasmids containing gene and flanking sequences can be maintained in most offspring in *P. caudatum*.

Previous studies of programmed genome rearrangements have suggested that IESs in ciliates might be degenerated remnants of transposons^70–73^. This hypothesis has been further strengthened by the discovery of domesticated transposases and their role in IES elimination in various ciliate species^36,74,75^. In *Paramecium tetraurelia* and *Tetrahymena thermophila*, domesticated *piggyBac* transposases (Pgm in *P. tetraurelia*; Tpb2p in *T. thermophila*) are responsible for DNA cleavage at IES ends^36,74,76,77^. Similarly, IES elimination in *Oxytricha trifallax* also requires the action of domesticated transposases, albeit from a different family^75,78^. In *P. caudatum*, we identified a Pgm protein homologous to Pgm in *P. tetraurelia*, which carries an intact catalytic triad (Fig. 4b and Supplementary Fig. 2). We demonstrated that Pgm localizes in the developing new MACs in the time and space in which DNA elimination occurs (Fig. 5a). Moreover, *PGM* RNAi during conjugation led to retention of IESs and strong lethality of sexual progeny (Fig. 4d, e), suggesting that *P. caudatum* Pgm plays an important role in IES elimination and the genome rearrangement process. In light of these findings, we suggest that Pgm is involved in DNA cleavage at IES ends during new MAC development.

It is worth noting that *P. caudatum* has an enormous germline genome and needs to eliminate 98% of its DNA during sexual development, including precise excision of IESs and imprecise excision of transposons^30^. Hence, whether there are other pathways besides Pgm-mediated DNA elimination that ensure the complete elimination of all germline-specific sequences in *P. caudatum* needs further investigation. In the future, detailed IES and transposon information can be obtained by sequencing the MIC genome. Additionally, sequencing of the new MAC genome and examining the extent of the IES retention observed in Pgm-silenced cells will allow us to determine whether Pgm is involved in the elimination of all germline-specific sequences, including transposons.

## Methods

### Cell culture

Two *P. caudatum* strains (5 and 6) of complementary mating types were kindly provided by Professor Xianyu Yang of Zhejiang A & F University (China). Cells were cultured at 25 °C in lettuce juice medium (5% fresh lettuce juice diluted in KDS solution)^79,80^ bacterized with *Klebsiella pneumoniae*.

### Silencing constructs for RNAi experiments

Open Reading Frame regions of *RPB1*, *ND7* and *PGM* genes of *P. caudatum* were retrieved from the Paramecium Database (https://paramecium.i2bc.paris-saclay.fr/)^42^. Fragments of *RPB1* (24–2426 nt), *ND7* (194–1271 nt) and *PGM* (1252–2658 nt) of both strains 5 and 6 were amplified using primers containing restriction enzyme sites for *SacI* and *Xhol* (Supplementary Table 2). We used the Paramecium Database to ensure that the fragments do not possess off-target possibilities in the MAC genome of *P. caudatum*. PCR fragments were purified by EasyPure Quick Gel Extraction Kit (Transgen Biotech, Beijing, China), digested with restriction enzymes *SacI* and *Xhol* (NEB, New England Biolabs, USA) and ligated into a *SacI/XhoI*-digested L4440 plasmid by T4 DNA ligase (NEB, New England Biolabs, USA) (Fig. 2a).

### RNAi by feeding during vegetative growth

Gene silencing was performed using a similar feeding method as previously described in *P. tetraurelia*, with the modifications described below^33^. Briefly, the silencing constructs were transformed into *Escherichia coli* HT115 (DE3) competent cells (Weidi Biotechnology, Shanghai, China), an RNase III-deficient strain with an isopropyl-β-D- thiogalactopyranoside (IPTG)-inducible T7 RNA polymerase. Bacteria were cultured in LB medium (supplemented with 100 µg/ml Ampicillin and 12.5 µg/ml Tetracycline) overnight at 37 °C. After overnight incubation, the precultures were diluted in LB medium to an OD_600_ of 0.04 and incubated for approximately 1.5 h under the same conditions, until the OD_600_ was between 0.07 and 0.1. dsRNA production was induced by the addition of 1 mM IPTG, and the cultures were incubated for at least 5 h at 37 °C to allow sufficient production of dsRNAs. Lastly, the *E. coli* cells were washed once with sterile lettuce juice medium, resuspended in lettuce juice medium supplemented with 166 µg/ml CaCl_2_ and adjusted to a final OD_600_ of approximately 0.12.

*P. caudatum* cells were washed twice with 10 mM Tris-HCl and seeded at a density of approximately 200 cells/ml into the silencing medium. An equal volume of fresh silencing medium was added to the cultures every day to ensure adequate bacterial supplies and continuous vegetative silencing. Gene silencing was performed in parallel in both strain 5 and 6. Control samples were fed HT115 bacteria with empty L4440 plasmids.

### Phenotypic analysis

Cells were observed under an Axio Imager D2 phase contrast microscope (Zeiss, Germany) and photographed using an Axiocam 506 color camera (Zeiss, Germany). To monitor the growth rate of cells after *RPB1* RNAi, the number of cells was measured every day before feeding for a period of seven days and compared with the control silencing (see Supplementary Data 1).

To evaluate the efficiency of *ND7* RNAi, cells were treated with 20% acetic acid to assess their ability of discharging trichocysts. By comparing with the control, cells were divided into three categories: almost all trichocysts discharged, a few trichocysts discharged, and no trichocyst discharge. The number of cells in each category were then calculated (see Supplementary Data 1).

### Reverse transcription quantitative PCR (RT-qPCR) analysis

Total RNA was extracted from cells after five days of continuous RNAi. Cells (approximately 6000 cells per sample) were pelleted by centrifugation (1000 *g* for 4 min) and resuspended in 1 ml of RNAprotect Cell Reagent (Qiagen, Hilden, Germany). RNA was extracted using the RNeasy Plus Mini kit (Qiagen, Hilden, Germany) and digested with DNase I (NEB, New England Biolabs, USA).

The RNA was reverse transcribed into single-stranded cDNA using HiScript III RT SuperMix for qPCR (Vazyme, Nanjing, China), following the manufacturer’s protocol. Quantitative PCR (qPCR) was performed using the QuantStudio 1 Real-Time PCR System (ThermoFisher Scientific, Waltham, MA, USA). Primers for qPCR were designed in regions outside of the dsRNA fragment (Fig. 3a, Supplementary Table 2). The small subunit rRNA gene, transcribed by RNA polymerase I, was selected as an internal control and Glyceraldehyde-3-phosphate dehydrogenase (*GAPDH*) was selected as a housekeeping gene, to assess the effect of *RPB1* RNAi. All reactions were performed in triplicate using ChamQ Universal SYBR qPCR Master Mix (Vazyme, Nanjing, China). The relative gene expression of target genes in RNAi and control samples was calculated using 2^−ΔΔCT^ method^81^ (see Supplementary Data 1) and statistical analysis was performed using the Fisher’s least significant difference (LSD) test in one-way analysis of variance (one-way ANOVA) by SPSS Statistics 25.0 software (IBM, New York, USA).

### RNAi by feeding during conjugation and survival test

The silencing bacteria were cultured in LB medium overnight at 37 °C, diluted in LB medium to an OD_600_ of 0.04 and incubated for approximately 1.5 h under the same conditions, until the OD_600_ was between 0.07 and 0.1. In contrast to the preparation of silencing medium used for vegetative growth, the bacteria were then centrifuged and resuspended in an equal amount of 5% lettuce juice medium (supplemented with 100 µg/ml Ampicillin and 166 µg/ml CaCl_2_). One mM IPTG was added to the bacterial cultures to induce dsRNA production, and the cultures incubated overnight at 37  °C.

As with RNAi performed during vegetative growth, two mating types of *P. caudatum* cells were washed and seeded into *PGM* silencing medium in parallel. Fresh silencing medium was added to the cultures every day. Control cultures were fed HT115 bacteria with empty L4440 plasmids.

After feeding with *PGM* silencing medium for 4 days, complementary mating types were starved and mixed to induce conjugation. For subsequent experiments, mating pairs were picked into mineral water after mixing. In each experiment, 24 mating pairs (4 h after mixing) or exconjugants (23 h after mixing) were picked and transferred individually into 0.5 ml fresh silencing medium or control medium (bacterized with *K. pneumoniae)* to monitor the growth of exconjugants daily. Five days after conjugation, the total number of sexual progeny was recorded for each condition. Cells were divided into three categories based on their cell numbers compared to the wild-type: healthy, normal growth; sick, slow-growing; dead, dead after less than three cell divisions (see Supplementary Data 1).

### Genomic DNA extraction and IES retention PCR

Total genomic DNA for IES retention PCR was extracted from around 10,000 exconjugants after RNAi using the DNeasy Blood & Tissue Kit (Qiagen, Hilden, Germany) following the manufacturer’s instructions. Taq master mix (Vazyme, Nanjing, China) was used for IES retention PCR with primers listed in Supplementary Table 1. The original uncropped agarose gel images for IES retention PCR can be seen in Supplementary Fig. 3.

### Construction of GFP and Flag-HA expression vectors

The open-reading frames (ORF) of the *RPB1* and *PGM* genes as well as putative regulatory regions (463 bp upstream and 476 bp downstream of the *RPB1* ORF; 437 bp upstream and 530 bp downstream of the *PGM* ORF) were amplified by PCR and cloned into the pGEM-T vector (Supplementary Table 3). Codon-optimized GFP was obtained from GenBank under the accession number AB071703^42^. GFP (723 bp) or Flag-HA (99 bp) was inserted after the start codon ATG by ligation of ends generated by the *BsaI* restriction enzyme (NEB, New England Biolabs, USA). The constructs were transformed into *E. coli* DH5α and extracted using the QIAGEN Plasmid Plus Midi Kit (Qiagen, Hilden, Germany).

### Microinjection and imaging

Constructs were linearized by the restriction enzyme *SapI* (NEB, New England Biolabs, USA), purified with phenol-chloroform and microinjected into the MAC of vegetative cells^48^. Injected cells were cultured in 1% culturing medium. For imaging, vegetative or conjugating cells were fixed in 4% paraformaldehyde fixation solution, washed thrice with 1× PBS, and stained with DAPI. Microscopy images were taken with an Axio Imager D2 microscope (Zeiss, Germany).

### Immunofluorescence and microscopy

Cells transformed with the Rpb1-Flag-HA construct were fixed in 4% paraformaldehyde in 1× PHEM at room temperature for 25 min. Then, cells were washed thrice with 1× PBS and permeabilized with 1% Triton X-100 in 1× PHEM for 1 h. After permeabilization, the cells were blocked in blocking solution (3% bovine serum albumin (BSA) and 1% Triton X-100 in TBSTEM) for 1 h and incubated with the primary antibody (HA-Tag (C29F4) Rabbit mAb, Cell Signaling Technology) at a dilution of 1:400 in blocking solution overnight at 4 °C. After three washes with blocking solution for 15 min, the cells were incubated with the secondary antibody (Alexa Fluor 546 Goat anti-rabbit IgG, Invitrogen) at a 1:4000 dilution in blocking solution for 45 min at room temperature. Lastly, the cells were washed with 1× PBS and stained with DAPI. Microscopy images were captured with an Axio Imager D2 microscope (Zeiss, Germany).

### Statistics and reproducibility

The LSD test in one-way analysis of variance (one-way ANOVA) was used to analyze the statistical significance of relative quantification between two samples in RT-qPCR analysis. A value of *p* <  0.01 was considered statistically significant. Asterisks indicate significant differences between expression level: **p  <  0.01. All RNAi experiments were performed three times respectively to examine reproducibility.

### Reporting summary

Further information on research design is available in the Nature Portfolio Reporting Summary linked to this article.

### Supplementary information

Supplementary Information

Description of Additional Supplementary Files

Supplementary Data 1

Reporting Summary

## Data Availability

All data are available in the article and its Supplementary Information. Numerical source data for all graphs can be found in Supplementary Data 1.
