# Supplementary material for: Application of RNA interference and protein localization to investigate housekeeping and developmentally regulated genes in the emerging model protozoan Paramecium caudatum
Source: Commun Biol. 2024 Feb 19;7:204. doi: 10.1038/s42003-024-05906-2 (PMC10876655; doi:10.1038/s42003-024-05906-2)
Supplement: Supplementary file 1 — Supplementary Information [file 42003_2024_5906_MOESM1_ESM.pdf]

Supplementary Information

Application of RNA interference and protein localization to investigate housekeeping and developmentally regulated genes in the emerging model protozoan *Paramecium caudatum*

Yunyi Gao[1], Therese Solberg[2,3], Rui Wang[1], Yueer Yu[1], Khaled A. S. Al-Rasheid[4], Feng Gao[1,5 *]

[1] *Key Laboratory of Evolution & Marine Biodiversity (Ministry of Education), and Institute of Evolution & Marine Biodiversity, Ocean University of China, Qingdao 266003, China*

[2] *Department of Molecular Biology, Keio University School of Medicine, Tokyo 160-8582, Japan*

[3] *Human Biology Microbiome Quantum Research Center (WPI-Bio2Q), Keio University, Tokyo 108-8345, Japan*

[4] *Zoology Department, College of Science, King Saud University, Riyadh 11451, Saudi Arabia*

[5] *Laoshan Laboratory, Qingdao 266237, China*

[*] Corresponding author, E-mail: gaof@ouc.edu.cn

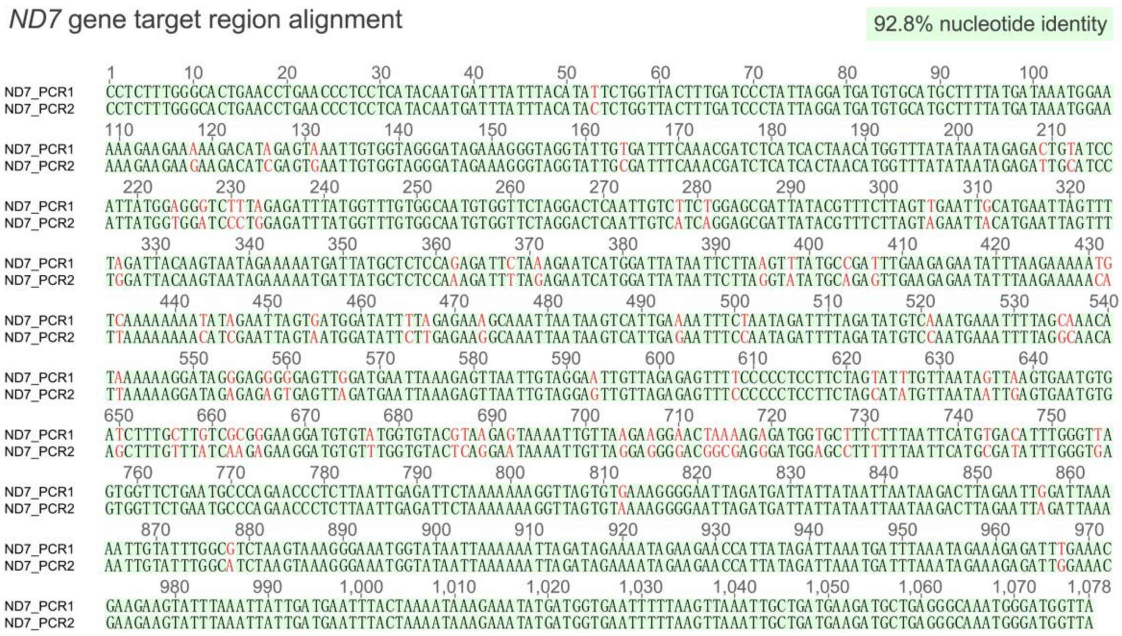

Supplementary Figure 1 Alignment of two *ND7* gene target regions. Alignment of nucleotide sequences of two distinct *ND7* gene sequences obtained by PCR amplification (ND7_PCR1, ND7_PCR2) performed by ClustalW. Green denotes identical nucleotides in the alignments. Red shows the different bases.

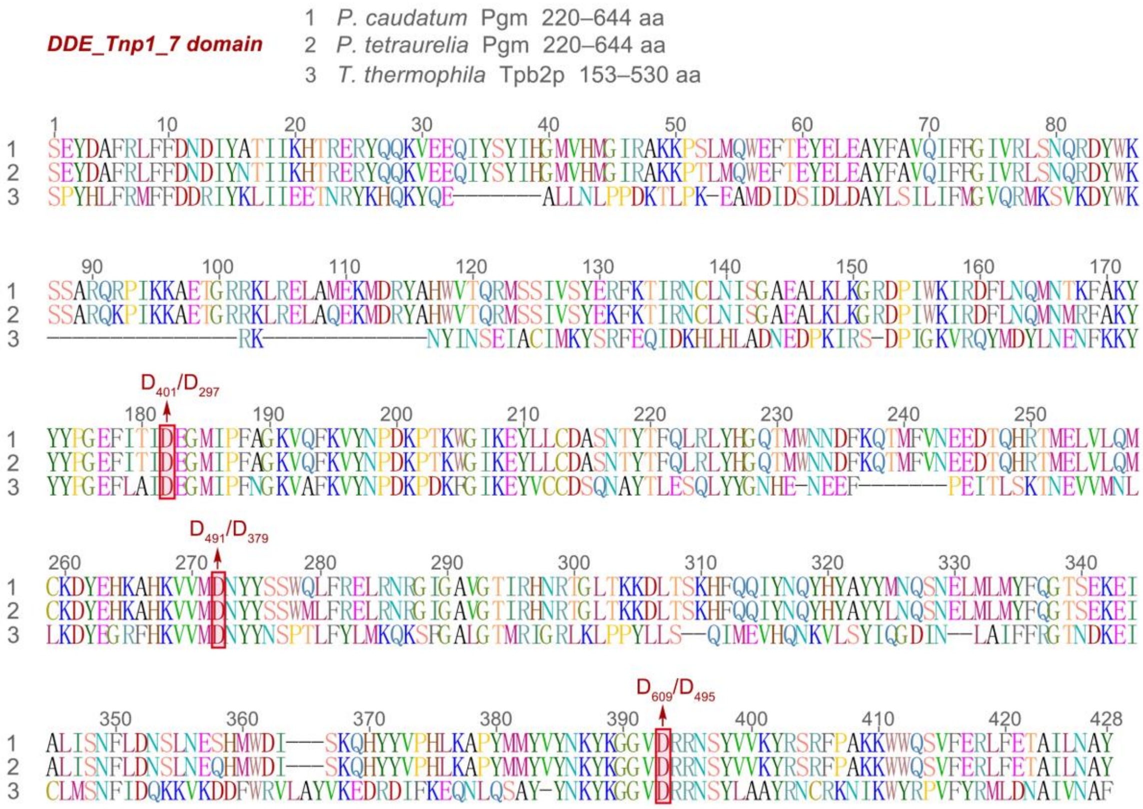

Supplementary Figure 2 Conservation of the DDD catalytic triad of domesticated *piggyBac* transposases in *Paramecium caudatum*, *Paramecium tetraurelia* and *Tetrahymena thermophila*. Three protein sequences of the DDE_Tnp_1_7 domain in *P. caudatum*, *P. tetraurelia* and *T. thermophila* were obtained by the Paramecium Database (https://paramecium.i2bc.paris-saclay.fr/) and Tetrahymena Genome Database (https://tet.ciliate.org/) and predicted by InterPro. Alignment of these sequences was performed by ClustalW. Conserved catalytic residues (D residues) are indicated by red frames.

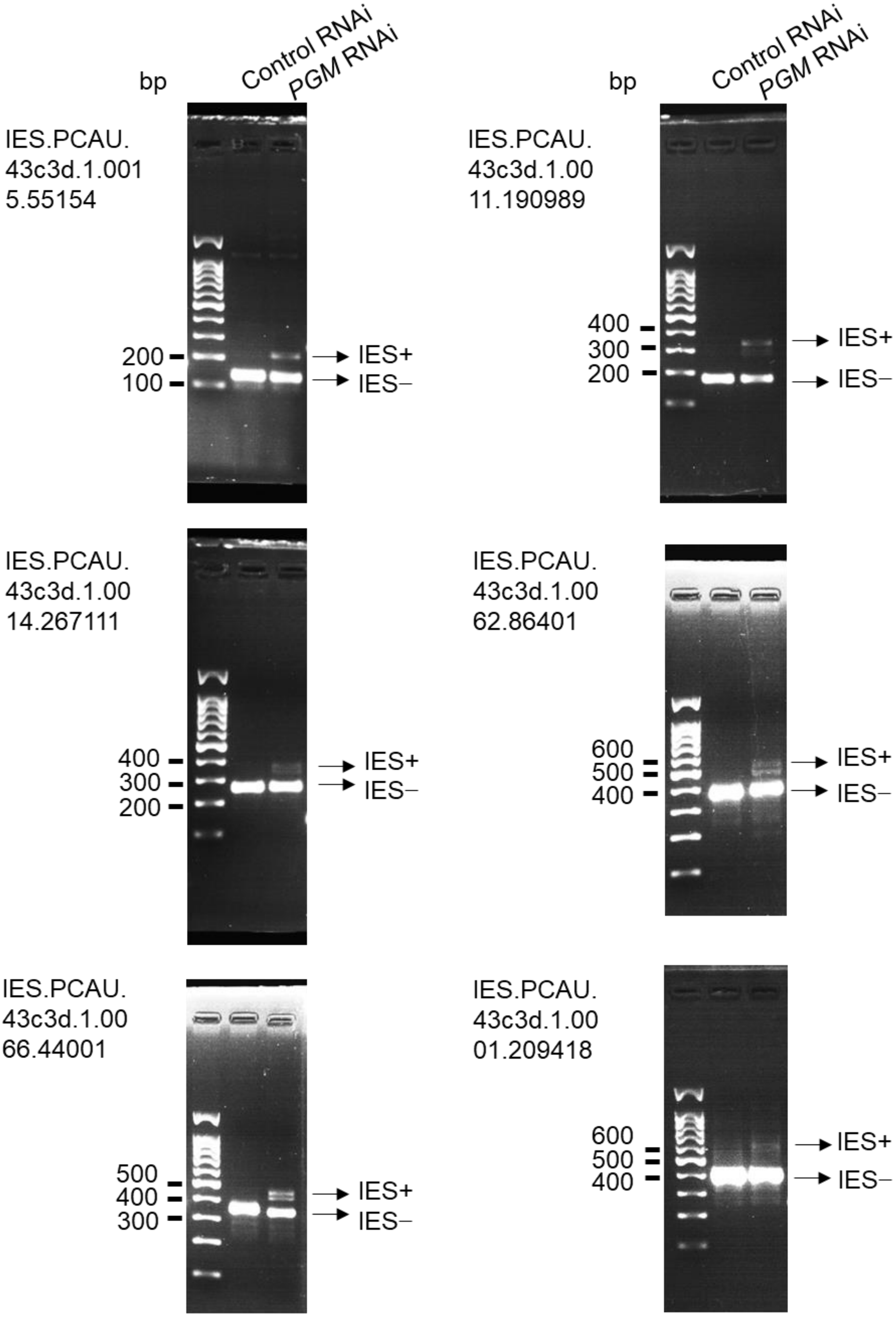

Supplementary Figure 3 The original uncropped agarose gel images for IES retention PCR. IES+: the size of bands with IES retention, IES－: the size of bands without IES retention.

Supplementary Table 1 IES information of *Paramecium caudatum* used in this study

IES ID	Size	Location	Primers 5' to 3'
IES.PCAU.43c3d.1.0015.55154	85	scaffold_0015:55154	F TTGTTTCAGAGGCTTGTTTA<br>R AGATTAGATCGGATGGATTG
IES.PCAU.43c3d.1.0014.267111	109	scaffold_0014:267111	F TTTGCTTGTCCTGGAGTTTC<br>R ATCCCACAGTTGATTTATCCTA
IES.PCAU.43c3d.1.0066.44001	118	scaffold_0066:44001	F TATGGGACAGTGTTTACCT<br>R AATACCCGTCGCAAATCTA
IES.PCAU.43c3d.1.0011.190989	154	scaffold_0011:190989	F TTTAGATATGCGTATGGAGA<br>R CCTATGATAAGTTTGGTTTG
IES.PCAU.43c3d.1.0062.86401	167	scaffold_0062:86401	F TATAGATCCCGATCCACAG<br>R CACTTAAATAAGCAATGCACATA
IES.PCAU.43c3d.1.0001.209418	285	scaffold_0001:209418	F GGGAATCTTGTCCTTGTCTA<br>R AGCCCAAAATAACAATGTAC

The IES information of *Paramecium caudatum* were retrieved from the Paramecium Database[40].

Supplementary Table 2 Oligonucleotides used in RNAi experiments

Primer name	5' to 3' Sequence	Use
RPB1_24_F	atatgagctcCACATACTCGTCAGCTCCAAT	PCR of *RPB1* for silencing
RPB1_2426_R	tttactcgagTGCTTCAGGTCCATAATCAAA	PCR of *RPB1* for silencing
ND7_194_F	atatgagctcCCTCTTTGGGCACTGAACCTG	PCR of *ND7* for silencing
ND7_1271_R	tttactcgagTAACCATCCCATTTGCCCTCA	PCR of *ND7* for silencing
PGM_1252_F	atatgagctcGGGTATGATTCCTTTTGCA	PCR of *PGM* for silencing
PGM_2658_R	tttactcgagTGAGTCTGGGATTAGTAGTT	PCR of *PGM* for silencing
RPB1_qPCR_3166_F	GTAGACATAGACTAACTCCAGAAGCATT	Primer of *RPB1* for qPCR analysis
RPB1_qPCR_3255_R	CTTCTCCAGGATGAGCCATA	Primer of *RPB1* for qPCR analysis
18S_qPCR_600_F	AGTCTATTGCTGTCTGGCTTCA	Primer of 18S for qPCR analysis
18S_qPCR_700_R	GGTAAATTGCCTACTCACCC	Primer of 18S for qPCR analysis
GAPDH_qPCR_899_F	TTGACGCTAAGGCTGGTA	Primer of *GAPDH* for qPCR analysis
GAPDH_qPCR_1005_R	GTGAATAGCCAAATCCAAA	Primer of *GAPDH* for qPCR analysis

The first six primers with restriction enzyme cutting site and protective base used to amplify the sequences from genomic samples. Uppercase letters indicate the gene sequences and lowercase letters indicate the restriction enzyme cutting site and protective base.

Supplementary Table 3 Oligonucleotides used in protein localization experiments

Primer name	5' to 3' Sequence	Use
RPB1_flankGFP_F	TAACTTTCCAATAAGAAGGATTC	PCR of *RPB1* for inserting pGEM-T vector
RPB1_flankGFP_R	AATAACTTCCGCATGACTC	PCR of *RPB1* for inserting pGEM-T vector
PGM_flankGFP_F	AAGAAATATGGTTTCGCTCCAA	PCR of *PGM* for inserting pGEM-T vector
PGM_flankGFP_R	CCTCCATGCGATAGAAAG	PCR of *PGM* for inserting pGEM-T vector
RPB1_BsaI_GFP_F	taggtctcgTCCAAGATAATCAGGCGCTGATTTCACATACTCG	Primer of *RPB1* for inserting GFP
RPB1_BsaI_GFP_R	taggtctcgTCATCATAATATATTTAATTAATCAAAATACTCACTTC	Primer of *RPB1* for inserting GFP
PGM_BsaI_GFP_F	taggtctcgTCCATATTTCAATGATGAAGAGGAAGAGGATTGGGGAGAC	Primer of *PGM* for inserting GFP
PGM_BsaI_GFP_R	taggtctcgTCATCATAAACTAAATCTATTCAATTCGATTATTTTCATTATGAATTCC	Primer of *PGM* for inserting GFP
Pc_GFP_F	taggtctcgATGAGAAAGGGAGAAGAATTGTTCACAGG	PCR of GFP for inserting
Pc_GFP_R	taggtctcgTGGAGCTCTCTTATACAATTCATCCATTCC	PCR of GFP for inserting
RPB1_BsaI_HA_F	taggtctcgTCATAGATAATCAGGCGCTGATTTCACATACTCG	Primer of *RPB1* for inserting Flag-HA
RPB1_BsaI_HA_R	taggtctcgCCATCATAATATATTTAATTAATCAAAATACTCACTTC	Primer of *RPB1* for inserting Flag-HA
Pc_FlagHA_F	taggtctcgATGATGGATTATAAAGATCATGATGG	PCR of Flag-HA for inserting
Pc_FlagHA_R	taggtctcgATGAGCATAATCTGGAACATCGTATGG	PCR of Flag-HA for inserting

The last ten primers with restriction enzyme cutting site and protective base used to amplify the sequences from genomic samples. Uppercase letters indicate the gene sequences and lowercase letters indicate the restriction enzyme cutting site and protective base.
