# Supplementary material for: Application of RNA interference and protein localization to investigate housekeeping and developmentally regulated genes in the emerging model protozoan Paramecium caudatum
Source: Commun Biol. 2024 Feb 19;7:204. doi: 10.1038/s42003-024-05906-2 (PMC10876655; doi:10.1038/s42003-024-05906-2)
Supplement: Supplementary file 4 — Reporting Summary [file 42003_2024_5906_MOESM4_ESM.pdf]

nature portfolio

Corresponding author(s): Feng Gao

Last updated by author(s): Jan 19, 2024

Reporting Summary

Nature Portfolio wishes to improve the reproducibility of the work that we publish. This form provides structure for consistency and transparency in reporting. For further information on Nature Portfolio policies, see our Editorial Policies and the Editorial Policy Checklist.

Statistics

For all statistical analyses, confirm that the following items are present in the figure legend, table legend, main text, or Methods section.

n/a	Confirmed	
☐	☒	The exact sample size (*n*) for each experimental group/condition, given as a discrete number and unit of measurement
☐	☒	A statement on whether measurements were taken from distinct samples or whether the same sample was measured repeatedly
☐	☒	The statistical test(s) used AND whether they are one- or two-sided<br>*Only common tests should be described solely by name; describe more complex techniques in the Methods section.*
☒	☐	A description of all covariates tested
☒	☐	A description of any assumptions or corrections, such as tests of normality and adjustment for multiple comparisons
☒	☐	A full description of the statistical parameters including central tendency (e.g. means) or other basic estimates (e.g. regression coefficient) AND variation (e.g. standard deviation) or associated estimates of uncertainty (e.g. confidence intervals)
☐	☒	For null hypothesis testing, the test statistic (e.g. F, t, r) with confidence intervals, effect sizes, degrees of freedom and P value noted<br>*Give P values as exact values whenever suitable.*
☒	☐	For Bayesian analysis, information on the choice of priors and Markov chain Monte Carlo settings
☒	☐	For hierarchical and complex designs, identification of the appropriate level for tests and full reporting of outcomes
☒	☐	Estimates of effect sizes (e.g. Cohen's *d*, Pearson's *r*), indicating how they were calculated

Our web collection on statistics for biologists contains articles on many of the points above.

Software and code

Policy information about availability of computer code

Data collection	no software was used
Data analysis	no software was used

For manuscripts utilizing custom algorithms or software that are central to the research but not yet described in published literature, software must be made available to editors and reviewers. We strongly encourage code deposition in a community repository (e.g. GitHub). See the Nature Portfolio guidelines for submitting code & software for further information.

Data

Policy information about availability of data

All manuscripts must include a data availability statement. This statement should provide the following information, where applicable:

- Accession codes, unique identifiers, or web links for publicly available datasets
- A description of any restrictions on data availability
- For clinical datasets or third party data, please ensure that the statement adheres to our policy

All data are available in the article and its Supplementary Information. Numerical source data for all graphs can be found in Supplementary Data. 1.

Human research participants

Policy information about studies involving human research participants and Sex and Gender in Research.

Reporting on sex and gender	NA
Population characteristics	NA
Recruitment	NA
Ethics oversight	NA

Note that full information on the approval of the study protocol must also be provided in the manuscript.

Field-specific reporting

Please select the one below that is the best fit for your research. If you are not sure, read the appropriate sections before making your selection.

☒ Life sciences ☐ Behavioural & social sciences ☐ Ecological, evolutionary & environmental sciences

For a reference copy of the document with all sections, see nature.com/documents/nr-reporting-summary-flat.pdf

Life sciences study design

All studies must disclose on these points even when the disclosure is negative.

Sample size	For RNAi experiments, we seeded at a density of approximately 200 cells/ml into the silencing medium. We took 10 samples (20 µl each) for each group and calculated the average of cell numbers to estimate the total cell numbers.
Data exclusions	No data were excluded from the analysis.
Replication	All RNAi experiments were performed three times respectively , and similar results obtained each time.
Randomization	In the same experiment, we initially inoculated cells cultured under identical conditions and ensured an equal cell seeding numbers in each sample.
Blinding	We did not use blinding for group allocation during the data collection.

Reporting for specific materials, systems and methods

We require information from authors about some types of materials, experimental systems and methods used in many studies. Here, indicate whether each material, system or method listed is relevant to your study. If you are not sure if a list item applies to your research, read the appropriate section before selecting a response.

Materials & experimental systems

n/a	Involved in the study	
☐	☒	Antibodies
☒	☐	Eukaryotic cell lines
☒	☐	Palaeontology and archaeology
☐	☒	Animals and other organisms
☒	☐	Clinical data
☒	☐	Dual use research of concern

Methods

n/a	Involved in the study	
☒	☐	ChIP-seq
☒	☐	Flow cytometry
☒	☐	MRI-based neuroimaging

Antibodies

Antibodies used	HA-Tag (C29F4) Rabbit mAb, Cell Signaling Technology, Cat# 3724, RRID: AB_1549585; Alexa Fluor 546 Goat Anti-Rabbit IgG Cross-Adsorbed Secondary Antibody, Invitrogen, Catalog # A-11071, RRID: AB_2534115.
Validation	HA primary antibody citation: Wang, C. D. et al. A small RNA-guided PRC2 complex eliminates DNA as an extreme form of transposon silencing. Cell Rep. 40, 111263 (2022).

Animals and other research organisms

Policy information about studies involving animals; ARRIVE guidelines recommended for reporting animal research, and Sex and Gender in Research

Laboratory animals	Paramecium caudatum, the single-celled protozoan, has been cultured in the lab for many years, which was kindly provided by Professor Xianyu Yang at Zhejiang A & F University (China). Cells were cultured at 25°C in lettuce juice medium.
Wild animals	The study did not involve wild animals.
Reporting on sex	Paramecium caudatum is single-celled protozoan. Sex is not relevant.
Field-collected samples	The study did not involve samples collected from the field.
Ethics oversight	Paramecium caudatum is single-celled protozoan, and does not require ethical approval.

Note that full information on the approval of the study protocol must also be provided in the manuscript.
